# Supplementary material for: Protracted development of stick tool use skills extends into adulthood in wild western chimpanzees
Source: PLoS Biol. 2024 May 7;22(5):e3002609. doi: 10.1371/journal.pbio.3002609 (PMC11075877; doi:10.1371/journal.pbio.3002609)
Supplement: S1 Table — (DOCX) [file pbio.3002609.s001.docx]

**Table S1**: Number of observations of hand grip used to hold stick tools recorded in the different contexts and age-class.

|  | AGE-CLASS | 1-2 | 3-4 | 5-9 | 10-14 | 15-19 | 20-29 | 30-39 | 40-49 | 50-54 |
| --- | --- | --- | --- | --- | --- | --- | --- | --- | --- | --- |
|  | Number of individuals | 11 | 10 | 14 | 9 | 17 | 13 | 4 | 3 | 1 |
| *Full hand grip* | Ant-dipping | 3 3 | 3  2 | -  - | -  - | -  - | 3  1 | -  - | -  - | -  - |
|  | Honey-dipping | 4 3 | - - | 1  1 | -  - | 1  1 | 1  1 | 6  2 | 1  1 | -  - |
|  | Insect-extraction | -  - | - - | -  - | -  - | 1  1 | -  - | -  - | -  - | -  - |
|  | Larvae extraction | - - | 1  1 | -  - | -  - | -  - | -  - | -  - | -  - | -  - |
|  | Nut extraction | 3 1 | - - | 1  1 | -  - | -  - | -  - | -  - | -  - | -  - |
| *Full hand thumb grip* | Ant-dipping | -  - | 22  7 | 7  5 | 15  3 | 1  1 | 6  2 | -  - | -  - | -  - |
|  | Honey-dipping | 6  3 | 3  1 | 12  3 | 2  1 | 24  10 | 5  3 | 1  1 | -  - | -  - |
|  | Insect-extraction | 7 1 | 3  2 | 19  3 | -  - | 2  1 | -  - | -  - | -  - | -  - |
|  | Larvae extraction | -  - | 18  1 | 32  6 | -  - | 15  5 | 2  1 | -  - | 2  1 | -  - |
|  | Nut extraction | -  - | - - | 22  2 | -  - | 3  2 | -  - | -  - | -  - | -  - |
|  | Termite mound perforation | -  - | - - | -  - | 14  5 | -  - | -  - | -  - | -  - | -  - |
| *Digits grip* | Ant-dipping | 2  2 | 12  5 | 45  10 | 29  11 | 63  16 | 182  21 | 4  2 | 20  2 | 4  1 |
|  | Bone marrow extraction | -  - | 8  1 | -  - | -  - | -  - | -  - | -  - | 77  7 | -  - |
|  | Honey-dipping | 1  1 | 6  4 | 69  7 | 7  1 | 249  22 | 95  11 | 51  8 | -  - | 70  5 |
|  | Insect-extraction | 1  1 | - - | 8  3 | 3  2 | 38  1 | 3  1 | -  - | -  - | -  - |
|  | Larvae extraction | - - | - - | 13 4 | 9  2 | 21  4 | 17  4 | -  - | 22  7 | -  - |
|  | Nut extraction | - - | - - | 6  1 | -  - | 28  3 | -  - | -  - | -  - | -  - |
|  | Termite mound perforation | - - | - - | -  - | 21  3 | -  - | -  - | -  - | -  - | -  - |
|  | Seed extraction | - - | - - | 4  1 | -  - | -  - | -  - | -  - | -  - | -  - |

*Note: The first row represent the number of occurrences and the second rows represent the number of videos*
