# Supplementary material for: Protracted development of stick tool use skills extends into adulthood in wild western chimpanzees
Source: PLoS Biol. 2024 May 7;22(5):e3002609. doi: 10.1371/journal.pbio.3002609 (PMC11075877; doi:10.1371/journal.pbio.3002609)
Supplement: S2 Table — (DOCX) [file pbio.3002609.s002.docx]

**Table S2**: Number of observations of tasks operated with stick tools recorded in the different age-class

| AGE-CLASS | 1-2 | 0-4 | 5-9 | 10-14 | 15-19 | 20-29 | 30-39 | 40-49 | 50-54 |
| --- | --- | --- | --- | --- | --- | --- | --- | --- | --- |
| Number of individuals | 11 | 10 | 14 | 9 | 17 | 13 | 4 | 3 | 1 |
| Insert | 38  13 | 76  20 | 239  36 | 100  22 | 446  51 | 314  38 | 62  10 | 122  16 | 74  6 |
| Prod | 41  11 | 35  12 | 84  31 | 57  16 | 226  33 | 229  34 | 22  7 | 88  11 | 71  5 |
| Screw | 3  1 | 3  2 | 12  2 | 15  5 | 6  2 | 5  4 | -  - | -  - | -  - |
| Stir | 7  5 | -  - | 6  6 | 3  3 | 43  19 | 43  8 | 4  4 | 4  4 | -  - |
| Lever | 7  5 | 12  2 | 53  12 | 21  5 | 73  17 | 17  4 | -  - | 28  11 | -  - |
| Pound | -  - | 10  6 | 12  7 | 1  1 | 107  19 | 17  5 | 27  7 | 2  2 | 1  1 |

*Note: The first row represent the number of occurrences and the second rows represent the number of videos*
