# Supplementary material for: Protracted development of stick tool use skills extends into adulthood in wild western chimpanzees
Source: PLoS Biol. 2024 May 7;22(5):e3002609. doi: 10.1371/journal.pbio.3002609 (PMC11075877; doi:10.1371/journal.pbio.3002609)
Supplement: S3 Table — (DOCX) [file pbio.3002609.s003.docx]

**Table S3**: Number of observations of actions operated with stick tools in the different context recorded in the different age-class

|  | AGE-CLASS | 1-2 | 3-4 | 5-9 | 10-14 | 15-19 | 20-29 | 30-39 | 40-49 | 50-54 |
| --- | --- | --- | --- | --- | --- | --- | --- | --- | --- | --- |
|  | Number of individuals | 11 | 10 | 14 | 9 | 17 | 13 | 4 | 3 | 1 |
| Ant-dipping | Insert | 5  5 | 37  10 | 52  12 | 44  11 | 64  16 | 191  21 | 4  2 | 20  2 | 4  4 |
|  | Lever | -  - | -  - | -  - | -  - | -  - | -  - | -  - | -  - | -  - |
|  | Prod | 22  7 | 14  6 | 44  11 | 37  9 | 49  13 | 172  21 | -  - | 20  2 | -  - |
|  | Screw | -  - | -  - | -  - | 1  1 | 2  1 | 1  1 | -  - | -  - | -  - |
|  | Stir | -  - | -  - | -  - | 1  1 | 4  3 | 8  3 | 1  1 | -  - | -  - |
|  | Pound | -  - | -  - | -  - | -  - | -  - | -  - | -  - | -  - | -  - |
| Bone marrow extraction | Insert | -  - | 8  1 | -  - | -  - | -  - | -  - | -  - | -  - | -  - |
|  | Lever | -  - | 4  1 | -  - | -  - | -  - | -  - | -  - | -  - | -  - |
|  | Prod | -  - | 7  1 | -  - | -  - | -  - | -  - | -  - | -  - | -  - |
|  | Screw | -  - | -  - | -  - | -  - | -  - | -  - | -  - | -  - | -  - |
|  | Stir | -  - | -  - | -  - | -  - | -  - | -  - | -  - | -  - | -  - |
|  | Pound | -  - | -  - | -  - | -  - | -  - | -  - | -  - | -  - | -  - |
| Honey-dipping | Insert | 11  5 | 9  5 | 82  8 | 9  2 | 274  23 | 101  11 | 58  8 | 78  7 | 70  5 |
|  | Lever | -  - | -  - | -  - | -  - | 6  5 | -  - | -  - | 4  4 | -  - |
|  | Prod | 1  1 | 3  2 | 14  7 | 9  2 | 165  16 | 51  10 | 22  7 | 65  6 | 71  5 |
|  | Screw | 3  1 | -  - | -  - | -  - | 4  1 | 3  2 | -  - | -  - | -  - |
|  | Stir | 5  4 | -  - | 2  2 | -  - | 38  15 | 27  5 | 3  3 | 4  4 | -  - |
|  | Pound | -  - | 8  5 | 3  3 | -  - | 73  18 | 17  5 | 27  7 | 2  2 | 1  1 |
| Beetle extraction | Insert | 11  5 | 3  2 | 27  6 | 3  2 | 41  1 | 3  1 | -  - | -  - | -  - |
|  | Lever | -  - | -  - | 2  2 | -  - | 4  1 | -  - | -  - | -  - | -  - |
|  | Prod | 1  1 | 2  2 | 14  6 | 2  1 | 7  1 | 4  1 | -  - | -  - | -  - |
|  | Screw | 3  1 | -  - | -  - | -  - | -  - | -  - | -  - | -  - | -  - |
|  | Stir | 5  4 | -  - | 3  3 | -  - | -  - | -  - | -  - | -  - | -  - |
|  | Pound | -  - | -  - | 9  4 | 1  1 | 34  1 | -  - | -  - | -  - | -  - |
| Larvae extraction | Insert | -  - | 19  2 | 45  6 | 9  2 | 36  7 | 19  5 | -  - | 24  7 | -  - |
|  | Lever | -  - | 8  1 | 20  6 | 5  1 | 33  7 | 17  4 | -  - | 24  7 | -  - |
|  | Prod | -  - | 9  1 | 8  4 | 3  2 | 4  2 | 2  2 | -  - | 3  3 | -  - |
|  | Screw | -  - | 3  2 | 12  2 | 1  1 | -  - | 1  1 | -  - | -  - | -  - |
|  | Stir | -  - | -  - | 1  1 | -  - | 1  1 | -  - | -  - | -  - | -  - |
|  | Pound | -  - | 2  1 | -  - | -  - | -  - | -  - | -  - | -  - | -  - |
| Nut kernel extraction | Insert | 3  1 | -  - | 29  3 | -  - | 31  4 | -  - | -  - | -  - | -  - |
|  | Lever | 1  1 | -  - | 29  3 | -  - | 30  4 | -  - | -  - | -  - | -  - |
|  | Prod | 2  1 | -  - | 2  2 | -  - | 1  1 | -  - | -  - | -  - | -  - |
|  | Screw | -  - | -  - | -  - | -  - | -  - | -  - | -  - | -  - | -  - |
|  | Stir | -  - | -  - | -  - | -  - | -  - | -  - | -  - | -  - | -  - |
|  | Pound | -  - | -  - | -  - | -  - | -  - | -  - | -  - | -  - | -  - |
| Termite fishing | Insert | -  - | -  - | -  - | 35  5 | -  - | -  - | -  - | -  - | -  - |
|  | Lever | -  - | -  - | -  - | 16  4 | -  - | -  - | -  - | -  - | -  - |
|  | Prod | -  - | -  - | -  - | 6  2 | -  - | -  - | -  - | -  - | -  - |
|  | Screw | -  - | -  - | -  - | 13  3 | -  - | -  - | -  - | -  - | -  - |
|  | Stir | -  - | -  - | -  - | 2  2 | -  - | -  - | -  - | -  - | -  - |
|  | Pound | -  - | -  - | -  - | -  - | -  - | -  - | -  - | -  - | -  - |
| Seeds from pod extraction | Insert | -  - | -  - | 4  1 | -  - | -  - | -  - | -  - | -  - | -  - |
|  | Lever | -  - | -  - | 2  1 | -  - | -  - | -  - | -  - | -  - | -  - |
|  | Prod | -  - | -  - | 2  1 | -  - | -  - | -  - | -  - | -  - | -  - |
|  | Screw | -  - | -  - | -  - | -  - | -  - | -  - | -  - | -  - | -  - |
|  | Stir | -  - | -  - | -  - | -  - | -  - | -  - | -  - | -  - | -  - |
|  | Pound | -  - | -  - | -  - | -  - | -  - | -  - | -  - | -  - | -  - |

*Note: The first row represent the number of occurrences and the second rows represent the number of videos*
