# Supplementary material for: Protracted development of stick tool use skills extends into adulthood in wild western chimpanzees
Source: PLoS Biol. 2024 May 7;22(5):e3002609. doi: 10.1371/journal.pbio.3002609 (PMC11075877; doi:10.1371/journal.pbio.3002609)
Supplement: S4 Table — (DOCX) [file pbio.3002609.s004.docx]

**Table S4**: Number of observations of stick use events and number of videos associated per individuals in the dataset

| Subject ID | Age observed | Number of stick use events | Number of videos |
| --- | --- | --- | --- |
| F1 | 5 | 23 | 5 |
| F2 | 17  20  21 | 109 | 13 |
| M1 | 20  21 | 70 | 7 |
| F3 | 2 | 1 | 1 |
| M2 | 14 | 5 | 3 |
| F4 | 7 | 16 | 4 |
| F5 | 29 | 3 | 1 |
| F6 | 14  15  16 | 21 | 6 |
| F7 | 1 | 1 | 1 |
| F8 | 17  18 | 53 | 10 |
| M3 | 20  21 | 13 | 2 |
| M4 | 3  4 | 11 | 6 |
| M5 | 38 | 4 | 1 |
| F9 | 13 | 7 | 1 |
| M6 | 13  16  19 | 37 | 5 |
| M7 | 2 | 7 | 3 |
| F10 | 45  49 | 21 | 3 |
| M8 | 3  5  8  9 | 31 | 8 |
| M9 | 15  16  17  21 | 48 | 5 |
| M10 | 4 | 4 | 1 |
| F11 | 45 | 77 | 6 |
| F12 | 5 | 6 | 1 |
| F13 | 3 | 1 | 1 |
| F14 | 23  24  25  27  28  29 | 40 | 7 |
| F15 | 22 | 3 | 1 |
| M11 | 19  20  23 | 127 | 13 |
| F16 | 1 | 1 | 1 |
| F17 | 5 | 9 | 2 |
| F18 | 16 | 13 | 1 |
| F19 | 5 | 1 | 1 |
| F20 | 15  16 | 43 | 2 |
| F21 | 2 | 11 | 2 |
| F22 | 16 | 5 | 1 |
| F23 | 36 | 4 | 2 |
| M12 | 2 | 1 | 1 |
| M13 | 4 | 2 | 1 |
| F24 | 7 | 6 | 1 |
| M14 | 23 | 4 | 2 |
| F25 | 15 | 2 | 1 |
| M15 | 9  11  14 | 19 | 7 |
| F26 | 4 | 18 | 1 |
| F27 | 5  6 | 30 | 3 |
| F28 | 16  20 | 74 | 7 |
| F29 | 43 | 24 | 7 |
| F30 | 6 | 19 | 3 |
| M16 | 1 | 1 | 1 |
| F31 | 22 | 6 | 1 |
| M17 | 19 | 36 | 7 |
| M18 | 1  5 | 3 | 2 |
| F32 | 13 | 4 | 1 |
| M19 | 2 | 1 | 1 |
| F33 | 16  19 | 8 | 2 |
| M20 | 10 | 3 | 1 |
| M21 | 17 | 12 | 1 |
| F34 | 2 | 2 | 1 |
| F35 | 35 | 3 | 2 |
| F36 | 7  8 | 32 | 4 |
| F37 | 4 | 1 | 1 |
| M22 | 3  5  8 | 72 | 5 |
| F38 | 50  51  54 | 74 | 6 |
| F39 | 3 | 3 | 1 |
| F40 | 8 | 12 | 4 |
| F41 | 24 | 30 | 5 |
| F42 | 18 | 8 | 1 |
| F43 | 25  26 | 3 | 2 |
| M23 | 12  13  14  17 | 48 | 8 |
| F44 | 14 | 6 | 1 |
| F45 | 15  17 | 5 | 3 |
| F46 | 31 | 51 | 8 |
| M24 | 1  4 | 11 | 2 |
