# Supplementary material for: Protracted development of stick tool use skills extends into adulthood in wild western chimpanzees
Source: PLoS Biol. 2024 May 7;22(5):e3002609. doi: 10.1371/journal.pbio.3002609 (PMC11075877; doi:10.1371/journal.pbio.3002609)
Supplement: S5 Table — Credible intervals of 89% and 95% are presented. All numeric predictor variables were standardized to mean = 0 and sd = 1. Values in bold represent credible intervals excluding zero. (DOCX) [file pbio.3002609.s005.docx]

**Table S5**. The effect of the hand grip type used, the age of the individual performing the task, its sex, and its group identity on the number of attempts to insert stick tools into high nutrients food holes (*Full hand thumb grip*^a^, Female^b^, and Group East^c^ as reference categories). Credible Intervals of 89% and 95% are presented. All numeric predictor variables were standardized to mean = 0 and sd = 1. Values in bold represent credible intervals excluding zero.

| Term | Estimate | SE | 89% CI | 95% CI |
| --- | --- | --- | --- | --- |
| Intercept | 0.32 | 0.17 | 0.05, 0.58 | -0.02, 0.64 |
| *Full hand grip*^a^ | 0.46 | 0.15 | **0.22, 0.69** | **0.17, 0.74** |
| *Digits grip*^a^ | 0.10 | 0.08 | -0.02, 0.22 | -0.05, 0.25 |
| Age | -0.10 | 0.07 | -0.21, 0.01 | -0.25, 0.05 |
| Age² | 0.05 | 0.06 | -0.03, 0.15 | -0.06, 0.19 |
| Sex^b^ | -0.08 | 0.07 | -0.19, 0.03 | -0.21, 0.06 |
| Group North^c^ | -0.02 | 0.20 | -0.34, 0.28 | -0.43, 0.35 |
| Group South^c^ | -0.04 | 0.11 | -0.22, 0.14 | -0.27, 0.18 |
