# Supplementary material for: Protracted development of stick tool use skills extends into adulthood in wild western chimpanzees
Source: PLoS Biol. 2024 May 7;22(5):e3002609. doi: 10.1371/journal.pbio.3002609 (PMC11075877; doi:10.1371/journal.pbio.3002609)
Supplement: S6 Table — Values in bold represent credible intervals excluding zero. (DOCX) [file pbio.3002609.s006.docx]

**Table S6**: Bayesian Regression model results of the effect of age on the probability of using the levering action in the larvae extraction context (Model 6A). Credible Intervals of 87%, 89% and 95% are presented. Values in bold represent credible intervals excluding zero.

| Term | Estimate | SE | 87% CI | 89% CI | 95% CI |
| --- | --- | --- | --- | --- | --- |
| Intercept | 0.09 | 0.91 | -1.33, 1.33 | -1.43, 1.42 | -1.87, 1.77 |
| Age | 0.87 | 0.67 | **0.01, 1.78** | -0.06, 1.86 | -0.47, 2.30 |
