# Supplementary material for: Protracted development of stick tool use skills extends into adulthood in wild western chimpanzees
Source: PLoS Biol. 2024 May 7;22(5):e3002609. doi: 10.1371/journal.pbio.3002609 (PMC11075877; doi:10.1371/journal.pbio.3002609)
Supplement: S7 Table — Values in bold represent credible intervals excluding zero. (DOCX) [file pbio.3002609.s007.docx]

**Table S7**: Bayesian Regression model results of the effect of age on the probability of using the levering action in the nut kernel extraction context (Model 6B). Credible Intervals of 85%, 89% and 95% are presented. Values in bold represent credible intervals excluding zero.

| Term | Estimate | SE | 85% CI | 89% CI | 95% CI |
| --- | --- | --- | --- | --- | --- |
| Intercept | 2.85 | 1.32 | 1.10, 4.66 | 0.83, 4.92 | 0.25, 5.80 |
| Age | 1.59 | 1.31 | **0.02, 3.40** | -0.18, 3.80 | -0.63, 4.73 |
