# Supplementary material for: Protracted development of stick tool use skills extends into adulthood in wild western chimpanzees
Source: PLoS Biol. 2024 May 7;22(5):e3002609. doi: 10.1371/journal.pbio.3002609 (PMC11075877; doi:10.1371/journal.pbio.3002609)
Supplement: S8 Table — (DOCX) [file pbio.3002609.s008.docx]

**Table S8**. Non-exhaustive table of the terminology used in the literature to describe different grips.

|  | Malherbe et al. | Feix et al. 2015 | Marzke et al 2014 | Bardo et al. 2017 | Costello and Fragasy 1988 | Spinozzi et al 2004 |
| --- | --- | --- | --- | --- | --- | --- |
| Species | Chimpanzees | Humans | Chimpanzees | Humans  Gorillas  Orangutan | Capuchins  Squirrel  monkey | Capuchins |
| 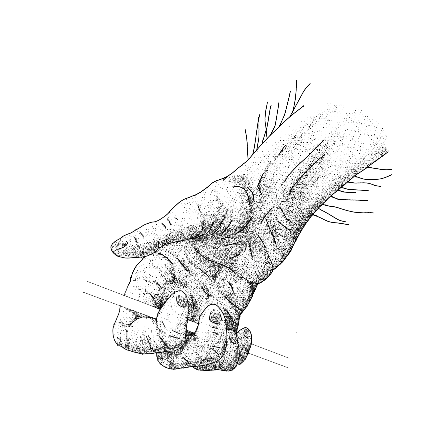 | Full hand grip | #1, #2 or #3 : Full hand wrap grasp | Transverse hook | Transverse  hook | - | Power grip |
| 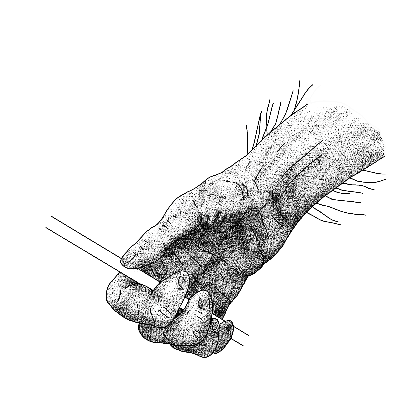 | Full hand thumb grip | #5 :  Light tool | - | - | - | - |
| 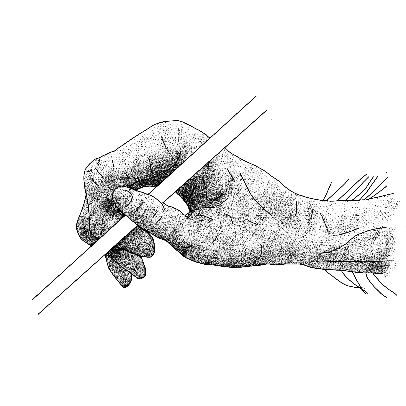 | Digits grip Category 1 | #16 :  Lateral grip | Two-jaw chuck pad-to-side | Two-jaw chuck pad-to-side | 1-2 Precision grip | I du-II dv |
| 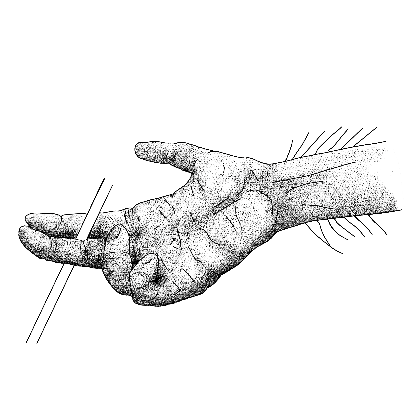 | Digits grip Category 2 | #23 : Adduction grip | Scissor hold | Scissor hold | 2-3 Precision grip | II du–III dr |
| 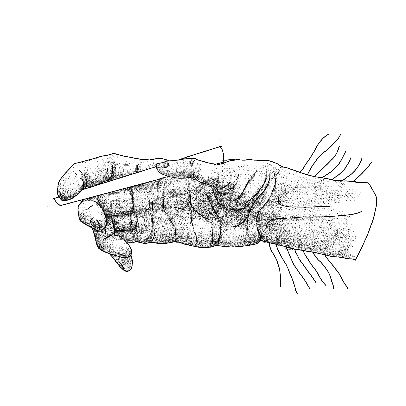 | Digits grip Category 3 | #21 : Tripod variation | - | - | 1-2,3 Precision grip | I du–II d, III d |
