# Supplementary material for: Protracted development of stick tool use skills extends into adulthood in wild western chimpanzees
Source: PLoS Biol. 2024 May 7;22(5):e3002609. doi: 10.1371/journal.pbio.3002609 (PMC11075877; doi:10.1371/journal.pbio.3002609)
Supplement: Attachment — Submitted filename: tool use review .docx [file pbio.3002609.s011.docx]

The authors present an unusual manuscript. There are very few studies that offer such a well- documented and detailed presentation of the ontogeny of tool use behavior. From that perspective, this is a refreshing and important addition to the literature.

However, there are three significant concerns that should be addressed. First, some sections require a much more thorough review of the literature. Second, there is an over-reliance on comparisons with the Corvid literature which must be addressed. Third, the assumptions about “cognition” are frequently vague and unconvincing. There are comments related to those concerns listed below.

The descriptions and documentation of the ontogeny of the tool behavior are the clear strengths in this paper. It can easily stand confidently on those data alone. The paper would be greatly improved if the assertions about cognition were removed. They are simply too vague and unconvincing, and they weaken the overall effort. In places, they seem to be inaccurate and misinformed.

I congratulate the authors on an excellent effort in major sections of the paper. In the case of this manuscript, less would certainly be much more in terms of the final product.

49-50 The authors have cited only partially quoted Beck’s (1980) definition of tool use. Also, that definition was revised in Shumaker et al. (2011). It would be preferable more accurate if the authors refer to the full and revised definition.

51-60 It is not clear how the authors draw the conclusion that tool use is typically differentiated into stereotyped and flexible tool use. While some authors have stated that, it is an overgeneralization as presented here. It would be reasonable to say some authors have suggested these labels. Hunt takes a fairly narrow view informed primarily from corvid tool use. But the idea that there are a “handful of birds and mammals” – presumably referring to species - that flexibly use tools is not well established or firmly documented. The authors should be clearer with this statement. While certainly true that some species demonstrate greater flexibility with tool use and manufacture the specifics are not well established.

64-81 The statements being made by the authors are not accurate or consistent with the literature on tool use. There are other species that habitually use tools flexibly. This section suggests that a more thorough review of the literature is essential, with a significant revision of the statements being offered. It also suggests that only the chimpanzee and corvid literature has been thoroughly reviewed, and that the literature on New Caledonian crows is being over emphasized and perhaps over interpreted.

82-108 As with the Introduction, the authors cite sources (primarily Byrne) that are presented as widely accepted regarding the relationship between tool use and cognition. That is not the case, and a more thorough review is required here. It is very fair to introduce these concepts, but that must be done accurately.

188-220 Very nicely done. The illustrations are very helpful.

335-338 The authors do a very good job offering detailed descriptions of the behaviors being documented. The suggested correlations to presumed cognitive complexity, or functions, are not well established or explained. These is pervasive throughout the manuscript.

683-732 The conclusions about chimpanzee cognition based on the Corvid model are tenuous and should be reconsidered. As mentioned in an earlier comment, there is an over reliance on Hunt throughout the manuscript and a broader literature review and overall perspective is essential.

749-750 This is a vague statement without citation. Chimpanzees and orangutans use all of the same modes of tool use in all settings. It is accurate to say that chimpanzees use more tools in combination with each other than other species except humans. However, the frequency of tool use in chimpanzees, or any other species, has not been documented. As a result, broad generalizations need to be made more cautiously.
