# Supplementary material for: Protracted development of stick tool use skills extends into adulthood in wild western chimpanzees
Source: PLoS Biol. 2024 May 7;22(5):e3002609. doi: 10.1371/journal.pbio.3002609 (PMC11075877; doi:10.1371/journal.pbio.3002609)
Supplement: Attachment — Submitted filename: Response to Reviewers - PBIOLOGY-D-23-03043R1.docx [file pbio.3002609.s012.docx]

**We are grateful to the editor for the opportunity to revise and resubmit our paper entitled “Prolonged development of stick tool use skills in wild western chimpanzees” (PBIOLOGY-D-23-03043R1). The comments made by the three reviewers, the academic editor, and the senior editor, whilst none of them are substantial, have nonetheless improved the coherence and impact of our work. We much appreciate this.**

**Following the feedback of the reviewers and editors, we further focused the key goal of the paper, which is to assess the protractedness of chimpanzees’ skill acquisition in stick tool use behavior, which we found extents into adulthood, supporting hominid evolution theories that relate prolonged offspring dependency to cognitive flexibility.**

**In the introduction we have streamlined the way we present the cognitive aspect of our work. We more carefully pin point some cognitive capacities considered necessary for stereotyped versus flexible tool use, such as reasoning, and working memory. We believe that introducing these capacities are important as they offer a complete background to our study. However, in the discussion, we no longer draw any conclusions about which specific cognitive mechanisms may explain the ontogenetic pattern observed in our results. We focus the conclusions on the most novel results – the highly protracted developmental processes needed to master more complex stick tool use behaviour.**

**Furthermore, following the advice of the academic editor and reviewer 2 we now provide a more comprehensive literature on flexible tool use behaviour across taxa (Lines 94-98).**

**Finally, in line with the academic editor’s suggestion, we now emphasize in the title the time point until which tool use continues to develop: “Protracted development of stick tool use skills into adulthood in wild western chimpanzees”.**

**We have also provided detailed responses (in bold) to the reviewers' comments in the next lines (line numbers refer to the clean version).**

REVIEWERS' COMMENTS:

**Reviewer #1:**

*This manuscript presents a high-quality and meticulously conducted and analysed study of the details of manual manipulation of tools through the lives of wild chimpanzees. It is clearly presented and sets the work in the context of a thorough review of the most relevant research literatures, including those concerning comparisons with humans and hominin evolution.*

*The analysis distinguishes the principal kinds of tool grips and their occurrence through ontogeny, demonstrating that the most finely skilled aspects of tool handling are not maturing until adulthood. This is a striking discovery, appropriate to this journal. I found figures 2 and 7, nicely portraying the numerical analyses of these changes over time, very clear and helpful. The findings will be of most immediate interest to others studying the anatomy, functional use and cognitive requirements of tool use in humans, earlier hominins and non-human primates, but also relevant to tool use, learning and culture more generally across humans and other animal taxa.*

*I have only a few more specific comments, amounting to suggestions for minor revision - by line number: -*

**We thank the reviewer for their positive feedback. We have made the changes according to their comments as presented hereafter.**

*68 - a reader may expect a reference here to the Rutz et al paper cited, on Hawaiian crows*.

**We have added the Rutz et al. (2016) paper on Hawaiian crows to this statement (Line 70).**

*Also note (and line 91) the recent discoveries of Goffins cockatoos using a tool set of three different tools - although this has been found in only a minority of their population studied:*

*O'Hara, M. et al. Wild Goffin's cockatoos flexibly manufacture and use tool sets. Curr Biol 2023.*

**We thank the reviewer for pointing it out and have now added this study as a further example of flexible tool use found in non-human animals (Line 97).**

*113 - precision grip - here I would think it may be expected to comment on the significant aspects of anatomy that are so different, which we do not read about until the Discussion.*

**We agree with the reviewer and have added a sentence to introduce the difference in hand anatomy between chimpanzees and humans. The sentence we added read as follow: “While significant differences exist between the hand anatomy of chimpanzees and humans, with the former having long digits and small and weak thumb considered inoperant in precise handling (Marzke, 1997)…” (Line 104-106).**

*Introduction - Mention of cultural learning is noticeably absent from the Introduction although references such as those by Biro, Musgrave and Whiten are focused on this. The Discussion picks up on this issue but I feel it ought to get at least a mention in the Introduction too.*

**Originally, we did not focus on cultural learning as we have no empirical data to confirm and identify which type of learning is involved in our study. Nonetheless, as we agree with the reviewer that this point is worthwhile mentioning, we now briefly discuss this in the introduction, in addition to the more detailed information provided in the discussion. Please see lines 102-104 where we added the following statement: “The ability to socially learn from others allows for material traditions and culture to exist between communities and populations (Boesch, 2003; Whiten et al., 1999)”.**

*175 - How many inter-observer tests were done?*

**Each observer was assigned to code 10% of the total video recorded time (please see lines 179-180). Overall the inter observer reliability tests results (using Cohen’s Kappa method) indicated good accuracy (>80%) of coding for both grip and action.**

*275 and 287 - Why the choice of 'age squared' rather than other functions?*

**We are not sure we fully understand this question from the reviewer, but we sought to answer it with the best of our understanding. We assume the reviewer is wondering why we have employed age square and not other terms. The age-squared term was added to the two models pointed out by the reviewer in order to model the non-linear pattern expected from our predictions shown in Table 2 (please see lines 350-351). Indeed, we predicted that with the switch between hand grips in model 1, the probability to use the full hand thumb grip will increase and then decrease. For model 2 we predicted that the young and old individuals will exhibit less motor control which we predicted would result in a decrease and then an increase in the number of attempts used through a chimpanzee’s life. Therefore, the “age-squared” term allowed to modelized the U or n shape expected from our predictions for both models.**

*707 - What is the nature of the evidence for social learning in these studies, in the wild? In captivity there is experimental evidence showing chimpanzees will acquire different forms of tool use to solve a foraging challenge through social learning, notably cultural transmission experiments by A Whiten et al (Nature 2005, Curr Biol 2007). The authors might consider citing these as adjuncts that support the circumstantial evidence from the wild.*

**Following a similar comment from reviewer 3 we have decided to remove the term “socially” from this sentence (line 697). We nonetheless thank the reviewer for the suggestion and have added the Whiten et al. papers to the manuscript (please see lines 701-702).**

*Minor 'typo level' points*

*Its not really my job to highlight these but here are a few noted: -*

*275 - grammar - delete 'A' ?*

*282 - apostrophe - grips'*

*300 - 'where we only included adult' better as 'where we included only adult …'*

*322 - 'confirm' ->  'confirmed' ?*

*512 - 'which increases' ->  'with increases' ?*

*553 - 'object' ->  ' objects' ?*

**Thank you. We made the changes accordingly.**

**Reviewer #2:**

*The authors present an unusual manuscript.  There are very few studies that offer such a well- documented and detailed presentation of the ontogeny of tool use behavior.  From that perspective, this is a refreshing and important addition to the literature.*

*However, there are three significant concerns that should be addressed.  First, some sections require a much more thorough review of the literature.  Second, there is an over-reliance on comparisons with the Corvid literature which must be addressed.  Third, the assumptions about "cognition" are frequently vague and unconvincing.  There are comments related to those concerns listed below.*

*The descriptions and documentation of the ontogeny of the tool behavior are the clear strengths in this paper.  It can easily stand confidently on those data alone.  The paper would be greatly improved if the assertions about cognition were removed.  They are simply too vague and unconvincing, and they weaken the overall effort.  In places, they seem to be inaccurate and misinformed.*

*I congratulate the authors on an excellent effort in major sections of the paper.  In the case of this manuscript, less would certainly be much more in terms of the final product.*

**We thank the reviewer for their constructive comments and positive feedback. We have revised the manuscript according to the reviewer’s comments and provide detailed responses in the following lines.**

*49-50  The authors have cited only partially quoted Beck's (1980) definition of tool use.  Also, that definition was revised in Shumaker et al. (2011).  It would be preferable more accurate if the authors refer to the full and revised definition.*

**We have followed the reviewer’s suggestion and provide a definition that refers also to Shumaker et al. (2011) definition. Since, we are specifically examining stick tool use behavior (i.e., an unattached object) we include a short but inclusive definition of tool use (please see lines 44-46). We believe that this definition is more relevant to our study:**

- **Full definition from Shumaker et al. (2011):
  “the external employment of an unattached or manipulable attached environmental object to alter more efficiently the form, position, or condition of another object, another organism, or the user itself, when the user holds and directly manipulates the tool during or prior to use and is responsible for the proper and effective orientation of the tool”.**
- **Definition now in our manuscript:
  “Tool use behavior, defined as the use of an environmental object to alter more efficiently the form, position, or condition of another object or organism (Beck, 1980; Shumaker et al., 2011)”**

*51-60  It is not clear how the authors draw the conclusion that tool use is typically differentiated into stereotyped and flexible tool use.  While some authors have stated that, it is an overgeneralization as presented here.  It would be reasonable to say some authors have suggested these labels.  Hunt takes a fairly narrow view informed primarily from corvid tool use.  But the idea that there are a "handful of birds and mammals" - presumably referring to species - that flexibly use tools is not well established or firmly documented.  The authors should be clearer with this statement.  While certainly true that some species demonstrate greater flexibility with tool use and manufacture the specifics are not well established.*

**We thank the reviewer for pointing out the need for us to be more general in our statement here in order to better reflect what is documented in the literature. We re-phrased the way we introduce the terms stereotyped and flexible tool use in order to not over-generalize these labels (please see lines 52-59). Additionally, we deleted the sentence stating that a “handful of birds and mammals are considered flexible tool users”.**

*64-81  The statements being made by the authors are not accurate or consistent with the literature on tool use.  There are other species that habitually use tools flexibly.  This section suggests that a more thorough review of the literature is essential, with a significant revision of the statements being offered.  It also suggests that only the chimpanzee and corvid literature has been thoroughly reviewed, and that the literature on New Caledonian crows is being over emphasized and perhaps over interpreted*

**We thank the reviewer for pointing out this oversight. We now provide a more detailed and accurate representation of flexible tool use across species and no longer focus on chimpanzees and New Caledonian crows (Lines 94-100).**

*82-108  As with the Introduction, the authors cite sources (primarily Byrne) that are presented as widely accepted regarding the relationship between tool use and cognition. That is not the case, and a more thorough review is required here.  It is very fair to introduce these concepts, but that must be done accurately.*

**We appreciate the reviewer’s comment and have made the necessary changes to the text. Specifically, we now provide a more detailed and comprehensive perspective on the topic of cognition and tool use by introducing the multiple cognitive concepts (e.g., reasoning, working memory, socio-cognitive capacities…) thought to be implicated in and possibly constraining flexible tool use behavior (Lines 83-93). We believe it is important to mention these concepts simply to offer a complete background to our study.**

*188-220  Very nicely done.  The illustrations are very helpful.*

**We thank the reviewer for this very nice comment.**

*335-338  The authors do a very good job offering detailed descriptions of the behaviors being documented.  The suggested correlations to presumed cognitive complexity, or functions, are not well established or explained.  These is pervasive throughout the manuscript.*

**We agree with the reviewer that caution is warranted when describing the cognitive underpinnings of these behaviors and their functions and have edited the text accordingly. Specifically, we now focus our description on the physical properties of the two tasks (i.e., nut kernel extraction and larvae extraction), explaining that the main difference between them is whether the food is visible or not (see lines 335-340).**

*683-732  The conclusions about chimpanzee cognition based on the Corvid model are tenuous and should be reconsidered.  As mentioned in an earlier comment, there is an over reliance on Hunt throughout the manuscript and a broader literature review and overall perspective is essential.*

**Following the reviewer’s constructive suggestion, we have revised the structure of this paragraph to offer the readers a greater focus on chimpanzees and our specific results. In particular, we now provide a greater emphasis on the relevance of our results to developmental literature, by discussing the prolonged juvenile dependency observed in chimpanzees and how it may be linked with the need to master the suitable actions needed for efficient extraction with stick tools. As also recognized by the reviewer and academic editor, the strength of this paper is our results showing the extensive time needed for chimpanzees to master the different tool use behaviors. We now put a greater emphasis on this result and focus less on the relation between tool use and cognition (please see lines 688-732).**

*749-750  This is a vague statement without citation.  Chimpanzees and orangutans use all of the same modes of tool use in all settings.  It is accurate to say that chimpanzees use more tools in combination with each other than other species except humans.  However, the frequency of tool use in chimpanzees, or any other species, has not been documented.  As a result, broad generalizations need to be made more cautiously.*

**We have edited and added a reference to our statement accordingly. The new statement is as follow: “Chimpanzees have one of the most extensive tool kit of non-human animals (Shumaker et al., 2011)” (Lines 749-750).**

**Reviewer #3:**

*This study describes the use of various techniques in the use of sticks in wild Western chimpanzees in a long term field site. I found the study well written and compelling, with very little to add or issues for the authors to address apart from the point below. Sadly I could not assess if and how previous comments by other reviewers have been addressed. In addition, I am also not really clear about the novelty brought by these results, which is not conveyed particularly well in the manuscript. What does it teach us beyond what is already in the literature, including in studies produced by several of the co-authors in this article?*

**We appreciate the reviewer’s valuable feedback and have made explicit efforts to highlight the unique contribution of our study to existing literature.**

**Our study of wild chimpanzees investigates the ontogenetic development of manual manipulation of stick tools, and that the associated stick action depends on the task. We find that acquiring the skills to use the most successful action per task can take until adulthood, especially for more complex tasks when food is hidden, even when the motor maturation required to enact the task occurs much earlier. This is especially valuable to our understanding of the constraints and drivers of tool use complexity and flexibility across taxa and the evolution of these traits in hominins.**

**Longitudinal studies on long-lived species, like chimpanzees, and across multiple tool use contexts have been lacking, and therefore our study also provides a unique perspective on this topic.**

*L706-710: The construction of this sentence is weird as it suggests that the references can refer to the first part of the sentence (that wild primates can SOCIALLY learn to choose tools with the right property). Yet, two of the cited papers are reviews, and the Sanz et al. paper does not show socially learn processes. To my knowledge, the only papers that could simultaneously sustain both points made by the authors regarding the socially learn use of the proper tools in wild primates would be the moss-sponge case described in wild chimps but even then, the studies would have to be combined to carry the point made (Hobaiter et al, 2014 Plos Bio, for social learning and Lamon et al. 2018 Proc Soc B for the right properties). But I may overlook other empirical studies.*

**We thank the reviewer for the comment. We have revised the statement and removed the term “socially” from the sentence (Line 697). We also added the Lamon et al. (2018) paper as a reference (Line 698).**
