# Supplementary material for: Protracted development of stick tool use skills extends into adulthood in wild western chimpanzees
Source: PLoS Biol. 2024 May 7;22(5):e3002609. doi: 10.1371/journal.pbio.3002609 (PMC11075877; doi:10.1371/journal.pbio.3002609)
Supplement: Attachment — Submitted filename: Response to Reviewer - PBIOLOGY-D-23-03043R2 - 270324.docx [file pbio.3002609.s013.docx]

REVIEWERS' COMMENTS:

**Reviewer #2**:

The authors have provided a detailed response to all reviewer comments along with a revised manuscript.

The authors have done an exceptionally good job of acknowledging all comments, editing the manuscript appropriately, and explaining those edits in their reply.  I am impressed with their collegiality and overall response to the feedback that was provided.

As a result, the manuscript is greatly improved and I strongly recommend publication.

I congratulate the authors on an excellent paper that will make an important contribution to the literature.  They would be very proud.

I also congratulate the editor and other reviewers. I believe this has been a model process for a manuscript review.

It has been a privilege to have been part of the review.  Thank you.

**We would like to thank the reviewer for the positive feedback on the changes we made to the manuscript. We believe that that comments made by the editors and the reviewers have helped improve the coherence of our findings. We much appreciate the effort made by all the editorial panel and would like to join the reviewer by thanking and acknowledging all involved in the review process of our paper.**
